# Non-adenomatous non-epithelial carcinoma (hemangiopericytoma) of prostate treated with conservative surgery followed by adjuvant chemoradiation

**DOI:** 10.3747/co.v16i4.408

**Published:** 2009-08

**Authors:** B. Chakrabarti, S.K. Ghosh, B. Basu, P. Gupta, S. Ghorai, S.G. Ray, C. Das

**Affiliations:** * Department of Radiotherapy, Calcutta Medical College, Kolkata, India

**Keywords:** Carcinoma, prostate, hemangiopericytoma, treatment

## Abstract

Hemangiopericytoma is a malignant vascular tumour of soft tissue. Microscopically, the tumour shows tightly packed cellular areas surrounding thin-walled branching blood vessels. Traditionally these tumours are treated using wide surgical excision. Only a very few cases of hemangiopericytoma of the prostate have been described worldwide. The feasibility of managing such a case with a combination of conservative surgery and adjuvant anti-malignancy treatment is unexplored.

Here, we report a case of hemangiopericytoma of the prostate treated with local excision, with preservation of prostate, followed by adjuvant radiotherapy (40 Gy in 20 fractions to pelvis followed by 24 Gy in 12 fractions as boost to prostate) and chemotherapy (doxorubicin and iphosphamide). Post-treatment computed tomography scan after 4 weeks showed regression of pelvic lymph nodes and a normal-appearing prostate. Levels of serum prostate-specific and carcinogenic embryonic antigen were normal throughout the period of treatment. To date, followup has been uneventful, except for occasional bouts of diarrhea.

We conclude that conservative surgery followed by adjuvant radiation and chemotherapy, with subsequent close follow-up, may adequately control localized disease in selected cases of hemangiopericytoma of the prostate. The role of conservative surgery in tumours located at other sites has yet to be defined.

## 1. INTRODUCTION

Hemangiopericytoma is a malignant vascular tumour of soft tissue. Microscopically, the tumour shows tightly packed cellular areas surrounding thin-walled branching blood vessels. Traditionally, these tumours are treated by wide surgical excision. Only a very few number of cases of hemangiopericytoma of the prostate have been described worldwide. Feasibility of managing such a case with a combination of conservative surgery and adjuvant anti-malignancy treatment is unexplored. Here, we report a case of hemangiopericytoma of the prostate treated with local excision, with preservation of prostate, followed by adjuvant radiotherapy and chemotherapy.

## 2. CASE DESCRIPTION

A 56-year-old man attended our oncology unit with a history of excision of a prostatic nodule about 6 weeks earlier. The patient initially attended a local doctor, reporting a history of increased frequency of micturition for 2 months and increased temperature for 1 month. Routine examination was normal, and urine culture was negative. The fever and pain subsided after treatment with antibiotics, but the increased frequency of micturition persisted. Digital rectal examination revealed a mildly enlarged nodular prostate, and pelvic sonography was done. A thick-walled nodule (38×36×35 mm) was detected in the prostate. A computed tomography scan was not done at that time. Serum prostate-specific antigen (psa) was 1 ng/mL. Cystoscopy showed no intravesicular lesions. Transrectal core needle biopsy diagnosed a probable case of alveolar rhabdomyosarcoma. The patient refused radical surgery, and only local excision of the prostatic nodule was done.

Histopathology of the excised nodule revealed a tumour composed of small vascular clefts and gaping vascular channels surrounded by spindle cells with round or oval plump nuclei. No hemorrhage or necrosis was seen. The tumour showed borderline atypia [4 of 10 high-power fields (4/10 hpf)]. Pathology margins for the excised prostatic nodule were negative. The tumour was diagnosed to be either a case of hemangiopericytoma, a solitary fibrous tumour, or a case of prostatic stromal proliferation of uncertain malignant potential. Immunohistochemistry found that the tumour cells expressed CD34, Bcl2 protein, Mic2, and vimentin, but were immunonegative for HMB45, synaptophysin, and chromogranin. Hence, a diagnosis of hemangiopericytoma was made.

Postoperative magnetic resonance imaging showed no residual prostatic nodule. Prostate was normal in size, but irregular in shape, and enlarged pelvic lymph nodes were reported on imaging. Image-guided biopsy of the nodes demonstrated spindle cells around branching vascular channels. Routine metastatic work-up, serum psa, and serum carcinogenic embryonic antigen (cea) levels were normal.

The patient refused any form of further surgery. Simple local excision of the prostatic nodule without any adjuvant treatment would not have sufficed, because positive pelvic nodes had been left behind. The patient was therefore given good supportive care and was treated postoperatively with external-beam radiotherapy followed by 6 cycles of adjuvant combination chemotherapy. External-beam radiotherapy was delivered using three-dimensional computerized treatment planning in a 3-field technique (1 anterior and 2 posterior oblique) delivering a dose of 40 Gy in 20 fractions to pelvis, followed by 24 Gy in 12 fractions as a boost to prostate, with adequate margin. Adjuvant combination chemotherapy delivered at intervals of 3 weeks consisted of doxorubicin 60 mg/m^2^ on day 1 and iphosphamide 2 g/m^2^ daily for 4 days, together with mesna and filgrastim support.

A ct scan 4 weeks after completion of treatment showed regression of the lymph nodes and a normal-appearing prostate. Serum psa and cea were normal throughout the period of treatment. Patient has been followed in our department for 42 months to the date of writing. He is asymptomatic, and follow-up has been uneventful, except for occasional bouts of diarrhea.

## 3. DISCUSSION

Hemangiopericytoma is an unusual perivascular tumour first described by Stout and Murray in 1942 1. It features uncontrolled proliferation of pericytes, which are cells described by Zimmermann in 1923 2,3. The tumour represents 2%–3% of all soft-tissue sarcomas in humans. It usually occurs in elderly patients during the 4th and 5th decades. The common sites are deep soft tissue, lower extremities, pelvis, and retroperitoneum 4. Head-and-neck lesions are also reported 5–9, and rare cases of primary hemangiopericytoma of bone may be found in pelvis, proximal femur, vertebrae, or humerus.

This tumour is generally rare in the urogenital system including the kidney; the other sites include the bladder, the prostate, and the spermatic cord 10–12. Hemangiopericytoma usually presents as a painless mass 4. Initial symptoms of the rare genitourinary tumours included hematuria, frequency of urination 11, and lower urinary tract obstruction 10.

Macroscopically, the tumour is soft and rubbery; microscopically, it is characterized by tightly packed small spindle cells surrounding thin-walled branching blood vessels (“stag horn” pattern). Microscopically, benign and malignant forms are distinguishable 11. Malignant forms are characterized by increased cellularity, prominent mitotic activity, and foci of necrosis or hemorrhage 4. Features indicating poor prognosis include increased cellularity, necrosis, hemorrhage, and mitotic figures exceeding 4/10 hpf 13. Tumours exhibiting nuclear atypia with more than 4/10 hpf may either recur or metastasize. Metastases are usually to lung and bone; lymph node metastasis is uncommon.

The spread pattern is principally hematogenous 2. Invasion of the bladder wall in urogenital tumours has been reported 10. Recurrence precedes metastasis in more than two thirds of patients with evidence of metastasis. The 10-year survival rate is 70%. The overall 5-year survival rate for microscopically dysplastic hemangiopericytoma is somewhat less than 50%.

The differential diagnosis of this lesion in prostate includes malignant fibrous histiocytoma, sarcomas, malignant peripheral nerve sheath tumour, and sarcomatoid carcinoma. Differentiation uses immunohistochemistry. Tissue immunohistochemistry shows immunoreactivity for vimentin (variable intensity), factor xiiia antigen, hla-dr antigen, and CD34. No staining or reaction with factor viii–related antigen, *Ulex europaeus* i lectin, α-smooth muscle actin, desmin, myoglobin, low- or high-molecular weight cytokeratin, or epithelial membrane antigen occurs.

Traditionally, these tumours are treated using surgical excision. Many authors have suggested that resectability may be the single most important determinant of clinical outcome 14. The role of adjuvant treatment is poorly defined. Use of adjuvant radiation therapy reduces the risk of local and distant recurrence 2.

Only a very few cases of hemangiopericytoma of the prostate have been described worldwide 15–17. Some patients were treated with transurethral resection of the prostate 10, but they later died of late recurrence in up to 50% of cases 2 and disseminated disease of the lung 3. The other patients were recommended to have early aggressive therapy and long-term follow-up 17. It was concluded that genitourinary tumours should be considered malignant and treated aggressively 12. The feasibility of managing such a case with a combination of conservative surgery and adjuvant anti-malignancy treatment had largely been unexplored.

## 4. CONCLUSIONS

Conservative surgery followed by adjuvant radiation and chemotherapy, and subsequent close follow-up may adequately control localized disease in selected cases of hemangiopericytoma of the prostate. The role of conservative surgery in such tumours at other sites has yet to be defined.
